# Study of a Controlled Piezoelectric Damper

**DOI:** 10.3390/s21103509

**Published:** 2021-05-18

**Authors:** Michał Makowski, Lech Knap

**Affiliations:** Institute of Vehicles and Construction Machinery Engineering, Warsaw University of Technology, ul. Narbutta 84, 02-524 Warsaw, Poland; lech.knap@pw.edu.pl

**Keywords:** piezoelectric damper, piezoelectric valve, mathematical model, identification of parameters, reduction of vibrations, experimental studies, numerical studies

## Abstract

In this work an original construction of a vibration damper controlled by means of a valve with a short time of operation lag is presented. The valve-controlling properties of the damper regulates the flow of fluid between the chambers of the damper and was constructed using piezoelectric actuators, whose characteristic feature is the possibility to change dimensions, e.g., length, under the influence of voltage. As a result, by changing voltage it is possible to control the throttle of the flow by changing the width of a gap, which influences a change of damping forces. Such a solution enables a quicker change of damping forces than in other kinds of controlled damper. Due to the obtained properties, the damper may be applied to reduce the vibrations of vehicles and machines that undergo quick-change loads. In the article, the results of experimental studies of the aforementioned damper are presented. Based on the results, dissipative characteristics were determined. Also, results of numerical studies comprising the development of a numerical model of a controlled piezoelectric damper are shown. Results of numerical studies, as well as experimental studies, are presented in the form of dissipative characteristics. Comparison of results of numerical and experimental studies confirms the possibility to apply this kind of construction in semi-active systems of vibration reduction of vehicles and machines.

## 1. Introduction

The topic of damping of vibrations in mechanical systems is current due to safety, the comfort of people and the protection of machines and their components. The existing solutions in terms of vibro-isolation are still being perfected, as well as new ones being developed. In the case of vehicles, the topic of controlled dampers is essential mainly due to driving characteristics, safety and comfort of passengers [1,2,3]. Many car manufacturers have already introduced or are introducing new solutions for vehicle suspensions. For example, Citroen developed the Hydractive III+ [4] system, where in order to reduce vibrations of the body controlled vibration dampers and actuators controlled by electromagnetic hydraulic valves were applied. Another example of solutions used to improve driving characteristics may be active roll stabilization developed by BMW [5] or E-Active body control developed by Daimler AG [6]. An interesting solution is applying electromagnetic valve to control the size of a gap and to change of ranges of damping forces in the Damp Tronic [7] solution by Bilstein or a CDC (Continuous Damping Control) damper by ZF Sachs [8].

In mechanical systems of machines, where there is a variable range of vibrations, it is often necessary to apply damping systems with variable damping characteristics [9], [10]. This is made possible by semi-active or active vibration dampers. In case of semi-active dampers, similarly as in dampers of vehicle suspensions, damping coefficient is changed during the operation of the system [11,12]. However, in the case of active systems of vibration reduction, additional energy used to react to variable load is supplied to the system [9,13]. Although active systems seem to be more effective, due to the necessity to supply significant energy to the system, semi-active systems are more often used [14,15,16].

In recent years, a significant part of research into controlled systems of vibration reduction concerned mainly dampers applied in semi-active systems, directly using possibilities to change the properties of a fluid. A characteristic feature of this type of device is the possibility to change damping forces based on a control signal from an electronic system, which creates an electromagnetic field influencing the properties of a fluid. This type of damper consists of dampers with magneto-rheological fluid (MR) or electro-rheological fluid (ER). A characteristic feature of magneto-rheological dampers is the change of damping forces together with the change of current intensity, which influences changes of magnetic field intensity acting on the fluid in the throttling gap. Studies of properties and modelling of an MR damper were shown, for instance, in the works [17,18]. In the case of dampers filled with electro-rheological fluid (ER), the value of damping forces is related to control of the value of voltage. Operation of electro-rheological dampers and systems of vibration damping with ER dampers were presented in the works [19,20,21].

Even though the aforementioned dampers have many advantages and are applied in chosen models of vehicles, these are still neither popular nor reliable solutions. Not only is it related to the costs of such dampers, but their durability stemming from frictional characteristics of rheological fluids and their segmentation as well. Other problems are those connected with controlling non-linear characteristics of dampers, as well as response time of these devices (on the level of 17–25 ms) [14,22]. The response time is excessively high, to the point that it requires prediction of road unevenness in front of a vehicle [6].

The piezoelectric materials are often used as sensors or actuators [23,24]. Piezoelectric materials have been used as actuators for the active vibration control of smart structures in [25]. The work [26] presents an adaptive active vibration control system with piezo actuator which is introduced to improve the isolation performance of an isolated structure. Piezo actuators can also be used to control pneumatic or hydraulic valves [27,28]. The work [29] presents the use of piezoelectric actuators to drive a micro pump.

This work presents a hydraulic damper, whose properties are varied by controlling fluid flow by a piezoelectric valve (PZ). The damper was filled with hydraulic oil, which prevents problems with e.g., delamination of fluid or excessive friction, as it may occur in the cases of MR and ER fluids. The PZ damper is also characterized by a possibility to vary damping forces which is related to directly controlling the size of the gap, through which oil flows between particular chambers of the damper during its operation. The size of the gap is controlled by a valve using a piezoelectric stack or actuator. A characteristic and advantageous feature of piezoelectric materials is the possibility to vary the executive element e.g., the length of a piezoelectric stack (PZ) under the influence of changes of control voltage. The changes occur very fast and, for instance, a manufacturer of piezoelectric stacks CEDRAT TECHNOLOGIES has on offer piezoelectric actuators acting with frequency of up to 7 kHz [30]. Exactly this type of actuator was used to develop the PZ valve of the PZ damper. For example, in the work [31] the results of studies using a PZ actuator to control damping coefficient of a shock absorber were shown.

In this article the construction and the results of numerical and experimental studies of the developed the PZ damper with the PZ valve were presented. Such a damper, after being produced, underwent studies of properties on a special laboratory stand. Based on results of these studies, dissipative characteristics were developed, depending on control signal, which in this case was voltage. In the work, a mathematical model of the PZ damper in the form of a rheological structure was proposed.

Based on results of experimental studies, an identification of parameters of a theoretical model of the PZ damper was performed. It allowed us also to conduct numerical studies of the application of the damper in a mechanical system of a vehicle suspension.

The results obtained from experimental and numerical studies confirmed the possibility to use controlled dampers with a piezoelectric valve to damp vibrations of vehicles and machines. This type of damper may be applied in systems, where quick changes of range of vibration frequencies occur. The presented solution may be competitive to the earlier mentioned MR and ER dampers due to use of hydraulic oil and shorter response time, which is on the level of approximately 9 ms (change of control signal in a full range from zero to maximum damping force). In particular, a piezoelectric damper may be applied to damp vibrations of a vehicle, which influences the safety and comfort of driving.

## 2. Construction of a Piezoelectric Valve (PZ) Damper

**Figure 1:**
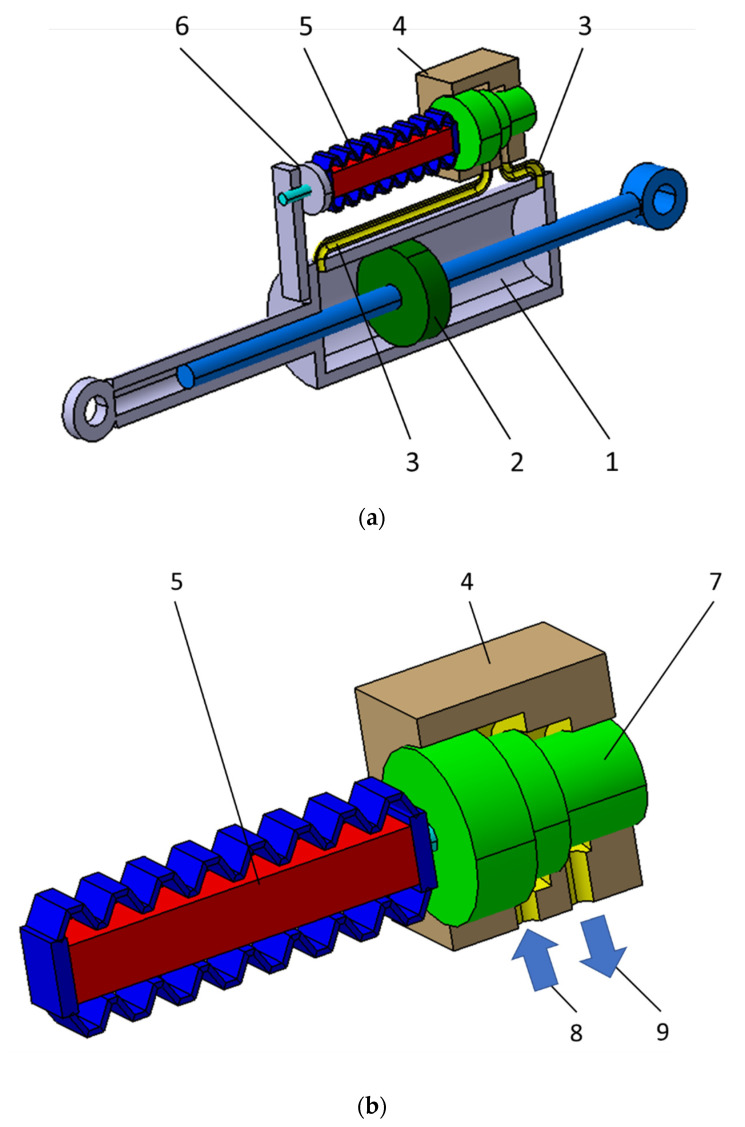
(**a**) Scheme of a piezoelectric (PZ) damper and (**b**) a piezoelectric valve.

The schematic construction of the developed PZ damper is shown in Figure 1a. In the hydraulic cylinder (1) there is hydraulic oil, which is forced through between the chambers of the cylinder by a moving piston (2).

Chambers of the hydraulic cylinder are tightly separated (seal on the piston), and the fluid flows through a duct (3), and next through the gap in the piezoelectric valve (4). As a result of fluid friction during the flow through the variable gap in the valve PZ (4) energy is dissipated, and its value depends on the size of the gap. The initial gap size is set with the adjusting screw (6).

Figure 1b shows a schematic construction of a piezoelectric valve used in the PZ damper. The gap in the PZ valve (1) is regulated by changing voltage in the electronic system powering the piezoelectric stack (5). Change of voltage is caused by change in position of the piston (7), which directly influences the change of the size of the gap of the hydraulic fluid flow and results in change of damping force. Arrows (8) and (9) show the direction of the fluid flow with the piston moving in one direction. With the piston moving in the reverse direction, the oil flow will be in reverse direction.

**Figure 2:**
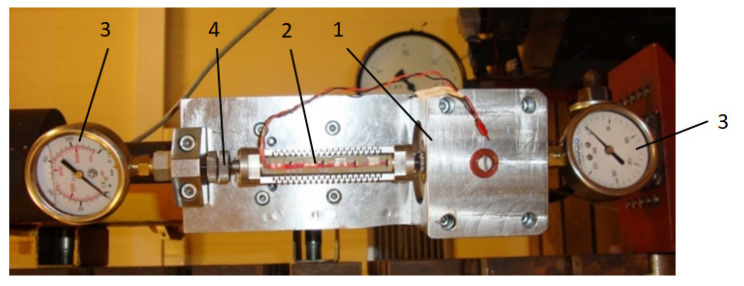
Piezoelectric damper.

The view of the actual construction of the test piezoelectric damper is shown in Figure 2. One may see the PZ valve block (1) connected to the piezoelectric stack (2) with PPA80L symbol, by Cedrat [30]. According to the manufacturer, the applied piezoelectric stack has a stroke of up to 85 μm max. The change of lengthening of the PZ stack influences the size of the cross-section area of the fluid flow, and this in turn enables obtaining adequate ranges of damping forces. It is necessary, however, to properly select the diameter to ensure the assumed flow resistances, which is not easy due to a small stroke realized by the piezoelectric stack.

Additionally, on the damper pressure sensors (3) were mounted, serving to control pressure, and indirectly to control forces acting on a piezoelectric element. The range of variability of damping forces is first regulated by the initial value of the gap in the valve obtained by means of a micrometer screw (4). This solution enabled establishing ranges of variables of damping forces depending on the needs of a particular damping system. Then, by changing the initial size of the gap, with the same displacements of the piston in the PZ valve it was possible to obtain different ranges of damping forces in the PZ damper, which enables different applications of the damper. The obtained functional characteristics of the PZ damper allow it to be used in systems of reducing vibrations, where due to the character of quick-change excitations it is necessary to quickly change damping forces. Such a necessity occurs in suspension of a vehicle, where excitations of vibrations depend on the profile of a road and the velocity of the vehicle. In such a case, kinematic excitations have a random character. Thus, in order to effectively damp vibrations of a vehicle it is necessary to constantly vary damping coefficient in a short time, which in turn enables different values of damping forces to be achieved.

The essence of possibilities of limiting vibrations of a mechanical system equipped with a PZ damper comes down to selection of adequate values of damping forces in the shortest time. Then, in the damper (mechanical system), by generating an adequate control signal of the piezoelectric stack, the size of the gap in the piezoelectric valve will change, which leads to a quick change of characteristics of the PZ damper. Quick control of a PZ damper also requires developing a simple and efficient mathematical model of the PZ damper. The numerical model developed and presented further in the work was used to conduct numerical studies in a vehicle model, where control signal was established based on the assumed algorithm. The construction of the damper and the numerical model developed and presented in the work may also be used to establish control algorithms of other kinds of dampers with variable characteristics e.g., MR and ER dampers. The control signal of vibration dampers may be determined based on different control criteria (e.g., criteria of limiting accelerations, limiting dynamic loads, or a mixed criterion combining two seemingly contradictory criteria). The solution to such a problem was more extensively discussed in the works [32,33].

## 3. Experimental Studies of a PZ Damper

**Figure 3:**
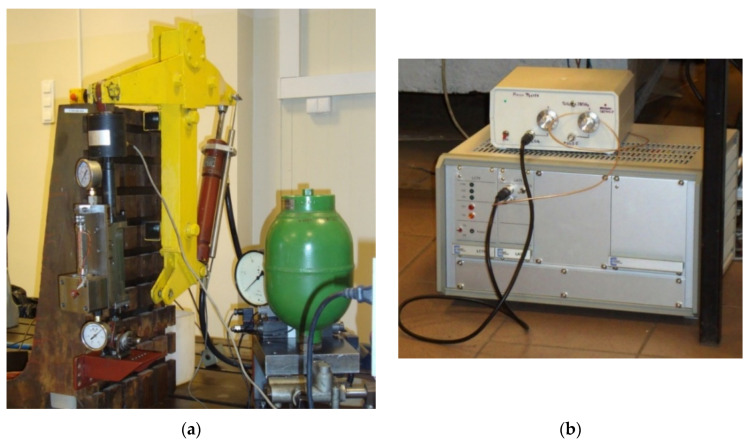
A stand to study properties of a PZ damper: (**a**) a general view of the stand, (**b**) electronic powering system of a PZ stack.

Experimental studies of properties of a piezoelectric damper were conducted on a test stand shown in Figure 3. Displacement of the PZ damper was realized by kinematic excitation realized by a hydraulic actuator (Figure 3a). The stand was equipped with displacement sensors and a force sensor. Measurement of these parameters was necessary to establish dissipative characteristics of the PZ damper. Signals from the sensors were registered by an electronic control-measurement system. The developed electronic system also served to control a hydraulic actuator, which realized a kinematic excitation of the damper. In order to power the PZ stack a dedicated electronic power supply shown in Figure 3b) was used. The system ensured voltage in the range 0–150 V and allowed energy to be partially retrieved during the change of focus of the piezoelectric element. The presented solution was introduced in order to decrease the demand for energy necessary to quickly refocus the PZ valve, which was obtained from the electric installation of a vehicle. In order to power a single PZ damper, it was necessary to supply power of 17 W. The change of voltage in the range 0–150 V enables lengthening the piezoelectric stack to the value of 85 μm when using PPA-80L actuator [34].

**Figure 4:**
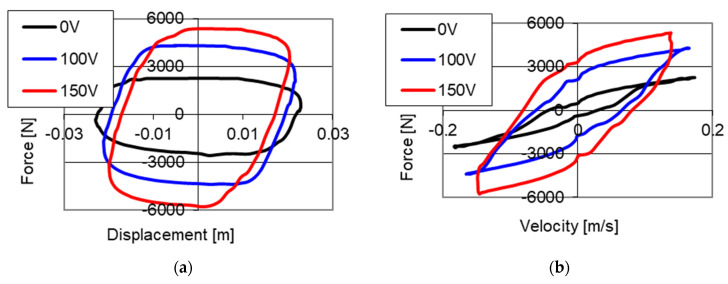
Results of experimental studies of a PZ damper without power supply (0 V) and with power supply 100 and 150 V with excitation with frequency 1 Hz and amplitude 0.022 m, initial value of the gap, (**a**) force–displacement, (**b**) force–velocity.

Results of experimental studies of the PZ damper are shown in Figure 4. The presented results were obtained with kinematic excitation with frequency 1 Hz and amplitude 0.022 m and with initial size of the gap on the level of 0.15 mm where the electronic system was without power supply (0 V) and was powered with 100 and 150 V respectively. The results are shown on two planes: force–displacement plane (hysteresis loop) and force–velocity plane (dissipative characteristics). On the graphs of force–displacement and force–velocity, one may notice a change of values of forces with increase on voltage in an electronic system.

Based on the presented results it may be concluded that with an increase of value of voltage (decrease in the size of the gap) the value of damping forces increases. It is related to the increase in friction during the fluid flow through a smaller gap in the valve. During the studies, a maximum value of damping forces of 2280 N without power supply to the PZ stack (0 V) was achieved, whereas with power supply 100 V the value of damping forces on the level of 4285 N was obtained, and with power supply 150 V (maximum value of voltage) a damping force with value of 5370 N was achieved. It was noticed that the increase of damping force is proportional to the increase of voltage powering the piezoelectric stack.

**Figure 5:**
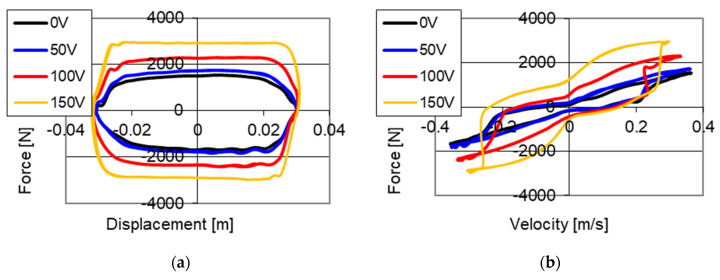
Results of experimental studies of a PZ damper without power supply (0 V) and with power supply 50, 100 and 150 V with excitation with frequency 2 Hz and amplitude 0.03 m, increased initial value of the gap, (**a**) force–displacement, (**b**) force–velocity.

Figure 5 shows results of experimental studies of the piezoelectric damper with kinematic excitation with a frequency of 2 Hz and amplitude 0.03 m. The studies were conducted with a changed base value of the gap by 0.1 mm, and then the size of the gap was 0.25 mm. Similarly, as in the case of previous studies, the values of power supply of the piezoelectric stack were changed in the range [0, 50, 100, 150] V. The results of the studies were also shown on the planes of force–displacement and force–velocity.

On the graphs (Figure 5) of force–displacement and force–velocity, one may notice the increase in values of damping forces together with the increase of voltage in the electronic system. In the case without power supply (0 V) a maximum value of forces 1514 N was obtained while with power supply 50 V force is equal to 1725 N, 227 N with voltage 100 V and 2940 N with power supply 150 V respectively. The increase of damping forces depending on voltage of power supply may be described using second degree dependency.

**Figure 6:**
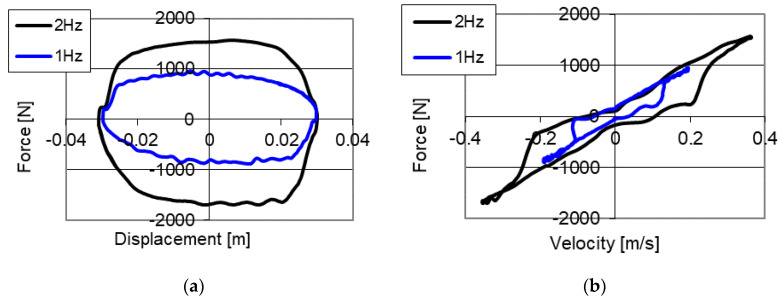
Results of experimental studies of a PZ damper without power supply (0 V) with excitation with frequency 1 and 2 Hz and amplitude 0.03 m, increased initial value of the gap, (**a**) force–displacement, (**b**) force–velocity.

Studies of the PZ damper without power supply to the piezoelectric stack with constant preset value of opening of the valve are shown in Figure 6. Changes of values of damping forces stem from changes of velocity of deformations realized by change of frequency of kinematic excitations, where the studies were conducted with excitation 1 and 2 Hz, and amplitude 0.03 m. As a result of studies, an increment of values of damping forces 870 N with excitation 1 Hz was obtained, increases to the value of 1530 N with excitation 2 Hz, and an increment of 43% of the initial value of opening of the valve.

Based on results of studies shown in Figure 4 and Figure 5 it may be concluded that the initial damping force (without power supply to the piezoelectric stack) is related to the initial cross section area in the PZ valve. Thus, the obtained values of damping forces are lower in Figure 5 in relation to the forces in Figure 4, because it arises from a greater initial gap in the PZ valve. This occurs despite increasing the velocity of deformations of the damper (as a result of increase of frequency of vibrations and amplitude of kinematic excitation), which usually increases damping forces (Figure 6).

Presented results of experimental studies also indicate the possibility to obtain change of damping force within the range of 200–250% with the same velocity of deformations of the damper, but different control signals of the PZ valve. Change of velocity of deformations of the PZ damper also influences an increment of damping forces by about 43% with a twofold increase of the velocity of deformations of the damper. However, in case of studies with the change of initial size of the gap in the piezoelectric valve, a fivefold increase of value of the initial force for different settings was achieved. This parameter was crucial in selecting the range of damping forces of the mechanical system. With application of the proposed solution, it is relatively easy to adjust the damping system to the range of vibration reduction. It is also possible not only by increasing the stroke of the valve, but also by increasing the diameter of the piston controlling the opening of the valve. Nevertheless, increasing the diameter is also related to the increase of the weight of the executive element, which may influence the decrease of velocity of the operation of the PZ valve. Thus, it is important to properly design the piston of the PZ valve, in order to achieve an adequate range of control of the damper with a short response time.

Studies of the influence of the change of temperature on the change of value of damping forces were also conducted. In order to perform such an analysis, the temperature of the external casing of the damper, which changed as a result of energy dissipation, was measured. The measurement was conducted using FLIR (Forward Looking Infrared) imaging camera.

**Figure 7:**
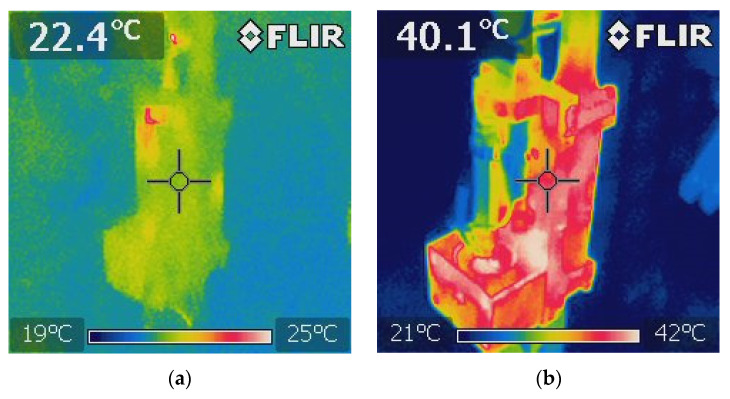
Measurement of the temperature of the damper during experimental studies, (**a**) initial phase of damper operation, (**b**) increased temperature of the damper after longer period of operation.

Figure 7 shows sample distribution of the area of temperature during damper operation. The damper was presented in an initial phase of operation (Figure 7a) and the damper after a longer period of operation (Figure 7b), where one may see almost a twofold change in values of temperature was also shown. Maximum value of temperature in Figure 7a is in the range of 25 °C, and the remaining part the temperature is close to ambient temperature 22 °C, whereas Figure 7b shows the maximum value of temperature 43 °C. Distribution of temperature areas on the surface of the PZ damper is related to places neighboring the chambers and ducts filled with oil. Then, in these places there are the highest values of temperatures, and areas with a lower temperature are surfaces of the piezoelectric stack and construction elements connected to its fixing.

**Figure 8:**
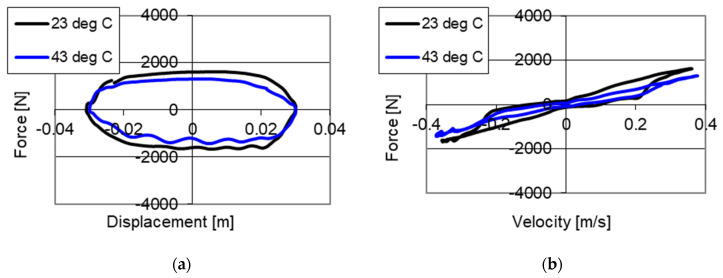
Results of experimental studies of a PZ damper without power supply (0 V) with excitation with frequency 2 Hz, amplitude 0.03 m with change of temperatures (**a**) force–displacement, (**b**) force–velocity.

**Figure 9:**
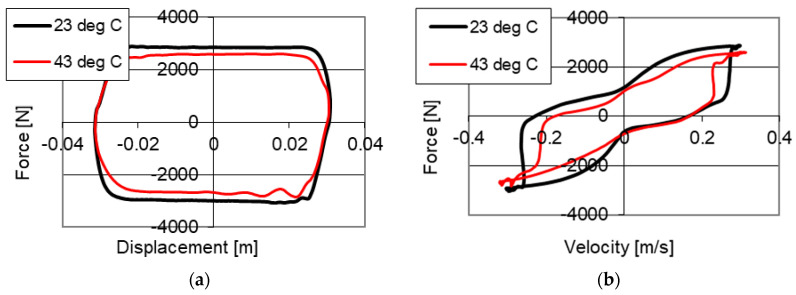
Results of experimental studies of a PZ damper with power supply (150 V) with excitation with frequency 2 Hz, amplitude 0.03 m with different temperatures (**a**) force–displacement, (**b**) force–velocity.

Results of studies of the PZ damper with a change of temperatures are shown in Figure 8 and Figure 9. The studies were conducted with kinematic excitation with frequency 2 Hz and amplitude 0.03 m. One may see curves in the force–displacement and force–velocity planes without power supply to the piezoelectric stack with two temperatures: 23 °C in an initial phase of operation and 43 °C, where the increase of temperature resulting from the operation of the damper may be observed.

Figure 8 shows results of studies of the PZ damper without power supply (0 V) with two temperatures. Figure 8a shows the changes of damping forces, where 1610 N was obtained with a temperature of 23 °C and a decrease of force to 1310 N with a temperature of 43 °C. Figure 8b shows the change of initial value of force from 170 N measured with temperature 23 °C, to the value of 75 N with the temperature of 43 °C. The change of damping forces is related to the change of fluid viscosity, where the studies were conducted with the same opening of the valve. As a result of the conducted studies a decrease of maximum damping forces by 300 N, with increased temperature of the fluid was achieved.

Studies of the influence of the change of temperature on the value of damping forces with power supply to the piezoelectric stack with value of 150 V are shown in Figure 9a. One may observe a change of values of damping forces with temperature increase. During the studies with a temperature of 23 °C, a maximum value of forces of 2900 N was obtained, and with 43 °C, 2580 N was achieved, respectively. The variation of damping forces resulted from the change of viscosity of the fluid, whereas in this case a decrease of maximum damping force by 320 N (approximately 10%) was obtained.

The studies conducted with the change of temperature indicate that the temperature increase influences decrease of values of damping forces, which was to be expected. The results obtained show that while controlling the dampers during experimental and numerical studies, the influence of temperature changes ought to be considered. In the initial phase of operation of the damper the values of forces, which may influence a greater value of dissipation of energy of vibrations, will be higher. The character of changes of damping forces in the case of temperature variation remains unchanged and difference only occur in values of damping forces. Thus, dissipative characteristics showing changes of damping forces with different temperatures are similar and may be described using the same mathematical model.

## 4. Identification of Parameters of a PZ Damper

Controlling properties of a PZ damper, and especially controlling damping force, requires developing adequate algorithms based on which, depending on input signal, control signals will be established. In the case of PZ dampers it is the value of voltage powering the piezoelectric element in the PZ valve. For proper and effective operation of a control algorithm, it is necessary to establish a numerical model of the damper, which may be used in control algorithms. It is also crucial to correctly identify parameters of the model of a damper. Thus, a proper model of a damper and its parameters are indispensable to conduct numerical studies of mechanical systems using controlled dampers and control algorithms.

In order to model controlled dampers, the mathematical models most often used are the Gamota–Filisko and Bouc–Wen [18,34,35] models. In the case of the Gamota–Filisko model, rheological structure is shown using five parameters, and in case of Bouc-Wen using seven parameters. In the case of the aforementioned models not all parameters directly reflect physical properties observed in experimental studies, and correct determination of parameters of these models is challenging. For example, in the work [35] parameters of a VPP (vacuum packed particles) damper were presented, and identified by means of the Bouc–Wen model. However, in the works [9,18] identification of a controlled MR damper using Gamota–Filisko and Bouc–Wen was shown.

**Figure 10:**
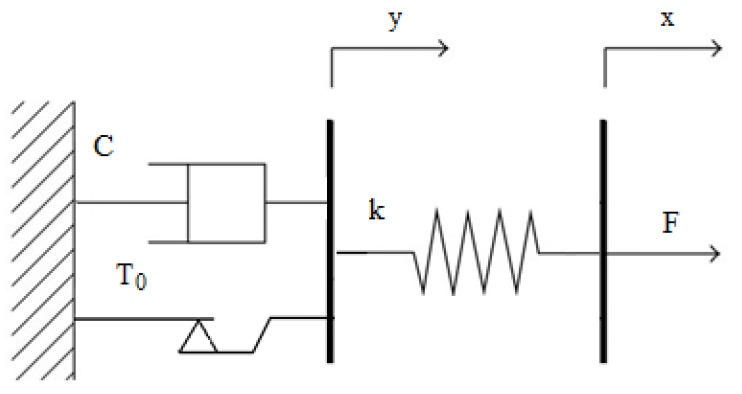
Rheological scheme of a PZ damper.

In this work, a model of a PZ damper was described in the form of a rheological structure by the Bingham model, which is shown in Figure 10. Viscoelastic properties of the damper are presented using three parameters, which may be identified based on experimental studies, which present respectively: $T_{0}$—dry friction, C—damping coefficient and k—stiffness coefficient. Thanks to limiting the number of parameters it is relatively easy to establish approximate values of parameters of the model of the damper based on dissipative characteristics showing changes of damping forces depending on the velocity of deformations of the damper. Such initially established parameters may serve as initial values of parameters of the model, which can be used during the next stage of identification with application of genetic algorithms or other nonlinear numerical optimization method. Such an approach enables a high compatibility of the results of experimental and numerical studies to be obtained based on the applied model of the PZ damper.

Mathematical model of forces acting on the structure in Figure 10 (so called Bingham model) is shown by the following simultaneous equations:

(1) $$F=\;k\left(x-y\right)$$

(2) $$C\;\dot{y}\;+{\tau T}_{0}=k\left(x-y\right)$$

(3) $$\tau =\left\{\begin{array}{c} \left\{\mathrm{sign}\;\dot{y}\;\right\},\\ \left[-1,+1\right], \end{array}\right.\begin{array}{c} \mathrm{when}\\ \mathrm{when} \end{array}\;\begin{array}{c} \;\dot{y}\;\neq 0\\ \;\dot{y}\;=0 \end{array}$$

where: $T_{0}$, C, k—parameters of the model, which are positive numbers characterizing viscoelastic features of the structure, x, y—coordinates of the model, F—force acting on the structure.

As already mentioned, initial identification of parameters based on results of experimental studies is merely the first stage of identification. In the second stage genetic algorithms were used, which are effective only when limitations are correctly defined. To evaluate the correctness of estimation of the parameters using genetic algorithms, a resulting convergence criterion was applied, based on minimizing the sum of square of differences of values of forces obtained during experimental and numerical studies. The criterion is shown by the following dependency:

(4) $$W_{S}=\;\min\left(\frac{\sum_{1}^{N}{\left(x_{\mathrm{pi}}-x_{\mathrm{si}}\right)}^{2}}{\sum_{1}^{N}x_{\mathrm{pi}}{}^{2}}\right),\;i=1,\ldots ,N$$

where: $x_{\mathrm{pi}}$—value of parameter obtained from experimental studies, $x_{\mathrm{si}}$—value of parameter obtained from estimation, N—number of samples.

Table 1 shows values of parameters of the model of the PZ damper obtained based on experimental studies, which were shown in Figure 4. Parameters of the model are related to the value of voltage powering the PZ stack, which is the control signal.

**Figure 11:**
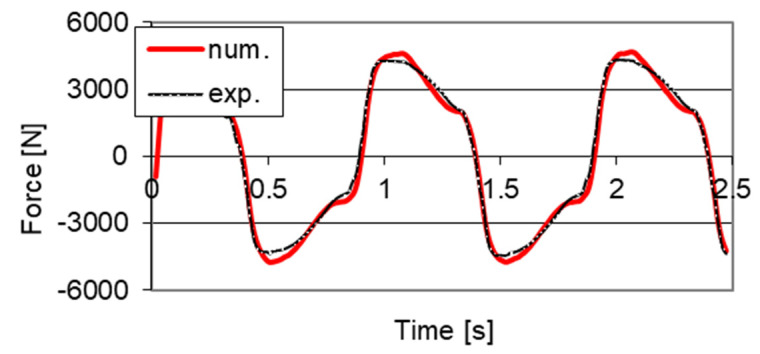
Changes of forces in time domain serving to estimate parameters of the model of the PZ damper.

**Figure 12:**
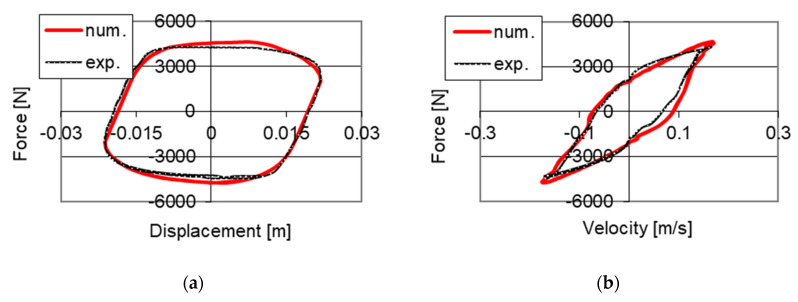
Comparison of results of numerical and experimental studies with kinematic excitation with frequency 1 Hz and amplitude 0.022 m, (**a**) force–displacement, (**b**) force–velocity.

The example of the obtained convergence of the developed numerical model and results of the mentioned experimental studies with powering the PZ stack with 100 V voltage were shown in Figure 11. One may observe the similarity of changes in time of the damping force obtained based on the proposed numerical model and results of experimental studies. However, Figure 12 shows the comparison of characteristics of the PZ damper on the planes of force–displacement and force–velocity obtained as a result of numerical simulations and experimental investigation.

**Figure 13:**
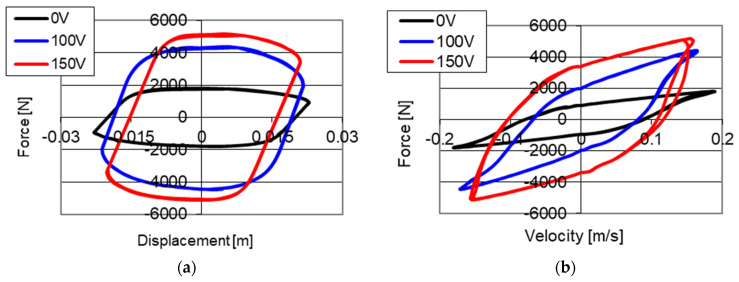
Results of numerical studies of the model of the PZ damper corresponding to power supply 0, 100, 150 V with kinematic excitation with frequency 1 Hz with amplitude 0.022 m with minimal opening of the gap, (**a**) force–displacement, (**b**) force–velocity.

Based on curves shown in Figure 11 and Figure 12, one may notice that a high convergence of solutions was obtained. Figure 13 additionally presents characteristics of the PZ damper established using the developed numerical model for different control voltages of the PZ valve 0 V, 100 V and 150 V, as well as excitations used during experimental studies presented in Figure 4.

The developed methodology of estimation of parameters of the PZ damper was also used to establish parameters of the model corresponding to results of experimental studies shown in Figure 5. The studies were conducted with the changed initial size of the gap and with kinematic excitation with frequency 2 Hz with amplitude 0.03 m. Table 2 shows parameters of the model of the damper obtained during studies at temperature 22 °C.

**Figure 14:**
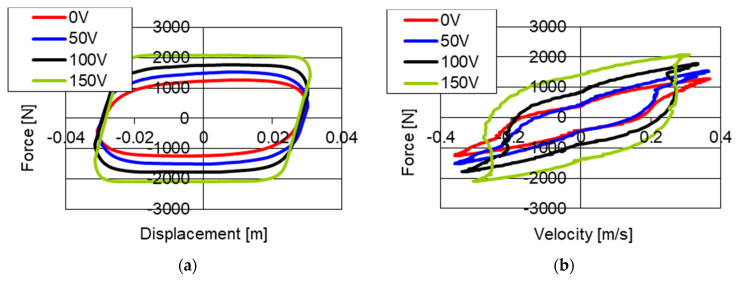
Results of numerical studies of the model of the PZ damper corresponding to voltage 0, 50 V, 100 V, 150 V with kinematic excitation with frequency 1 Hz with amplitude 0.022 m with change of size of the gap, (**a**) force–displacement, (**b**) force–velocity.

Similarly as in the previous case all parameters of the model of the damper change together with the changes of voltage powering the PZ valve. In the greatest extent, the change of voltage influences the value of dry friction force. The value of dry friction force shown in Table 2 changes 4 times, and the other parameters change in a smaller range. Based on the conducted identification of parameters, the characteristics of the damper shown in Figure 14 were developed.

Experimental studies indicated that characteristics of the PZ damper changes together with temperature change. Thus, identification of parameters of the damper was also performed based on studies conducted with a temperature increase to 43 °C. Values of parameters obtained in the process of identification are shown in Table 3.

One may observe a drop of the values of parameters representing dry friction ($T_{0}$). For example, with power 0 V the value of friction amounts to 301 N at 23 °C and drops to 169 N at 43 °C. At the same time it is evident that with the voltage increase, there is decrease in value of friction forces obtained for different temperatures. With power supply of 150 V, the greatest drop in values of dry friction from 1325 N at 23 °C to 1078 N at 43 °C was obtained. Based on the conducted analysis it may be concluded that the change of parameter of dry friction is greatest in the damper without power supply.

**Figure 15:**
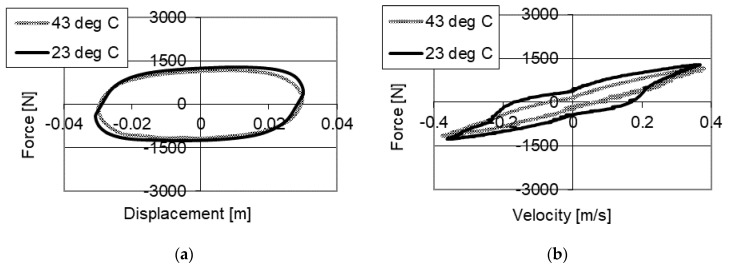
Results of numerical studies of the model of the PZ damper of the influence of temperature on changes of damping forces corresponding to studies without power supply to the PZ stack, (**a**) force–displacement, (**b**) force–velocity.

**Figure 16:**
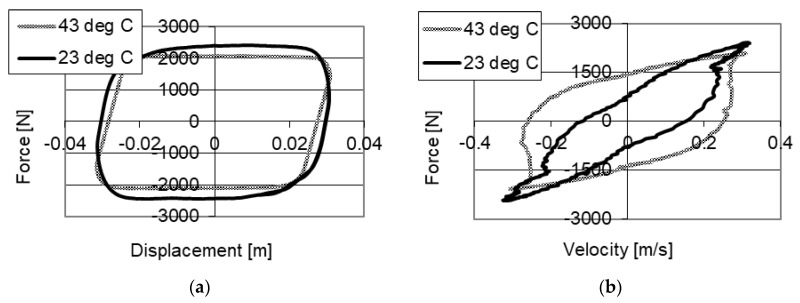
Results of numerical studies of the model of the PZ damper of the influence of temperature on changes of damping forces corresponding to studies with power supply to the PZ stack with voltage 150 V, (**a**) force–displacement, (**b**) force–velocity.

Figure 15 and Figure 16 show characteristics of the PZ damper obtained as a result of conducted numerical studies based on the identification of parameters. Figure 15 shows the results of studies conducted without power supply to the PZ stack of the damper, where two curves are visible—at the temperature of 23 °C and 43 °C. On the characteristics one may see the decrease in damping forces. Similarly, the influence of temperature on damping forces is visible in Figure 16, where studies were conducted with power supply voltage of 150 V. The differences obtained are in a similar range, as in case of experimental studies (Figure 8 and Figure 9).

## 5. Numerical Studies of a Vehicle with PZ Dampers

**Figure 17:**
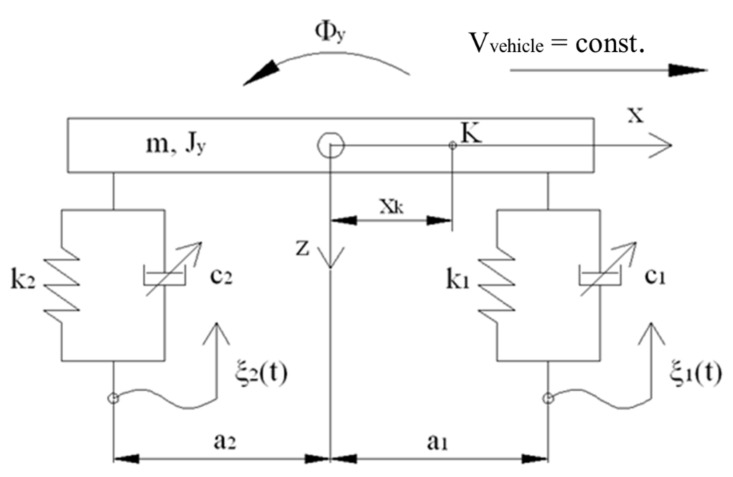
Model of the vehicle.

Controlled PZ dampers were used to conduct numerical studies of the model of a vehicle. The value of damping forces in the system was established based on algorithm with the assumed comfort criterion. Based on the algorithm friction force was established in dampers for every moment in time. In order to conduct studies the criterion of minimizing the module of friction forces was assumed. The studies were conducted on a simplified flat model of the vehicle shown in Figure 17. The vibrating system characterizes the body with mass m and inertia $J_{y}\;$and forces acting on springs S and damper T. Vibrations were kinematically extorted ξ with the simplification of lack of separation of the wheels from the surface, which is typical of vehicles moving on roads. Based on displacements of the body representing the body of the vehicle and excitations, the deflection of the suspension U, as well as velocity of deformations of the suspension V were established. The model was described in the coordinates:

(5) $$X={\left[z,{\;\Phi }_{y}\right]}^{T}$$

The model of the vehicle is shown by the following equation:

(6) $$M\ddot{X}+H\left(S+T\right)=0$$

where $M=\mathrm{diag}\;\left(m,J_{y}\right)$.

In the Equation (6) weigh was omitted, and calculations were conducted from the position of balance. In the equation vector H determines the space of configuration of operation of forces S and T, which is determined in the form of:

(7) $$H_{1}={\left[1,-a_{1}\right]}^{T},{\;H}_{2}={\left[1,{\;a}_{2}\right]}^{T}$$

The value of damping forces during studies was established based on the assumed algorithm, where vector T is determined as follows:

(8) $$T=\gamma (H^{T}X,{\;H}^{T}\;\dot{X}\;)$$

where γ is the operator describing algorithm of determination of control signals, which is dependent on deflections and velocities of the deformation of suspension.

During the studies it was assumed that accelerations will be minimalized in the chosen point (K) on the body of the vehicle (Figure 17). The value of the acceleration is described using the dependency:

(9) $$a_{K}=-G^{T}M^{-1}H\left(S+T\right)=a_{0}+D^{T}T$$

where $a_{0}$—acceleration in the center of gravity, vector G is in the form of $G={\left[1,{\;x}_{k}\right]}^{T}$.

The comfort indicator, depending on forces in dampers in every moment in time is in the form of:

(10) $$K\left(T\right)=\left|a_{0}+D^{T}T\right|$$

The measure of quality of the indicator is maximum comfort, hence the smallest value of the indicator K is selected. The set of results of friction forces depends on the velocity of deformations V, which is established in the set of solutions of permitted forces $\Omega \left(V\right)$.

(11) $$\Omega \left(V\right)∶=\left\{T\in R^{2}:T_{i}\in w\left(V_{i}\right),\;i=1,2\right\}$$

**Figure 18:**
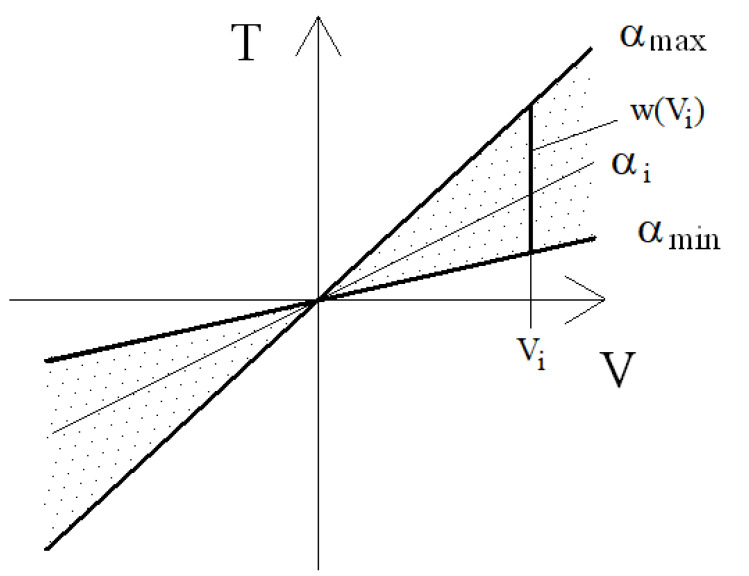
PZ damper characteristics.

Friction force in a single damper $T_{i}$ is established based on characteristics of the PZ damper (Figure 18) and control signal $w\left(V_{i}\right)\;$ at an established velocity of deformations. The values of friction forces depend on parameters describing the control system of the valve, and the field of control range is schematically shown in Figure 18. The set of solutions is limited by signals (α_min_, α_max_) showing values of permitted voltage powering the piezoelectric stack (0–150 V). Regarding the characteristics, the set of solutions is shown, where based on the control signal α_i_, with the preset speed $\;V_{i}$ the friction speed of a single damper $\;T_{i}\;$ is established, where i signifies the number of a single wheel of the vehicle. The presented characteristics were developed based on experimental studies. Based on these characteristics, the value of voltage powering the piezoelectric stack is established, which serves to determine mathematical parameters of the PZ damper shown in Figure 10.

Based on dependencies (10) and (11), the optimization problem of friction forces is developed. The solution to the problem is in the form of:

(12) $$T_{K}\in \underset{T\in \Omega \left(V\right)}{\mathrm{Arg}\;\min}K\left(T\right)$$

A more detailed description of the method of establishing vectors of damping forces was described in the works [32,33].

The work presents sample numerical studies of a vehicle with controlled damping forces and with an assumed comfort criterion. The model of the vehicle was characterized by the following parameters: weight of the body of the vehicle m = 1200 kg, stiffness of the spring k_1_ = k_2_ = 47,500 N/m. For comparative purposes studies were also conducted with a constant damping forces coefficient c = const., where the value of damping coefficient was assumed c_1_ = c_2_ = 4500 Ns/m. In order to realize the optimization problem limits of the damping coefficient were defined c_min_ = 750 Ns/m, c_max_ = 45,000 Ns/m, which are related to the limits of voltages powering the piezoelectric stack with voltage U_min_ i U_max_. Simulations were conducted with kinematic excitation by sinusoidal harmonic function with amplitude 0.02 m, velocity V_vehicle_ = 90 km/h and frequency of excitation 2.25 Hz and 4.5 Hz.

**Figure 19:**
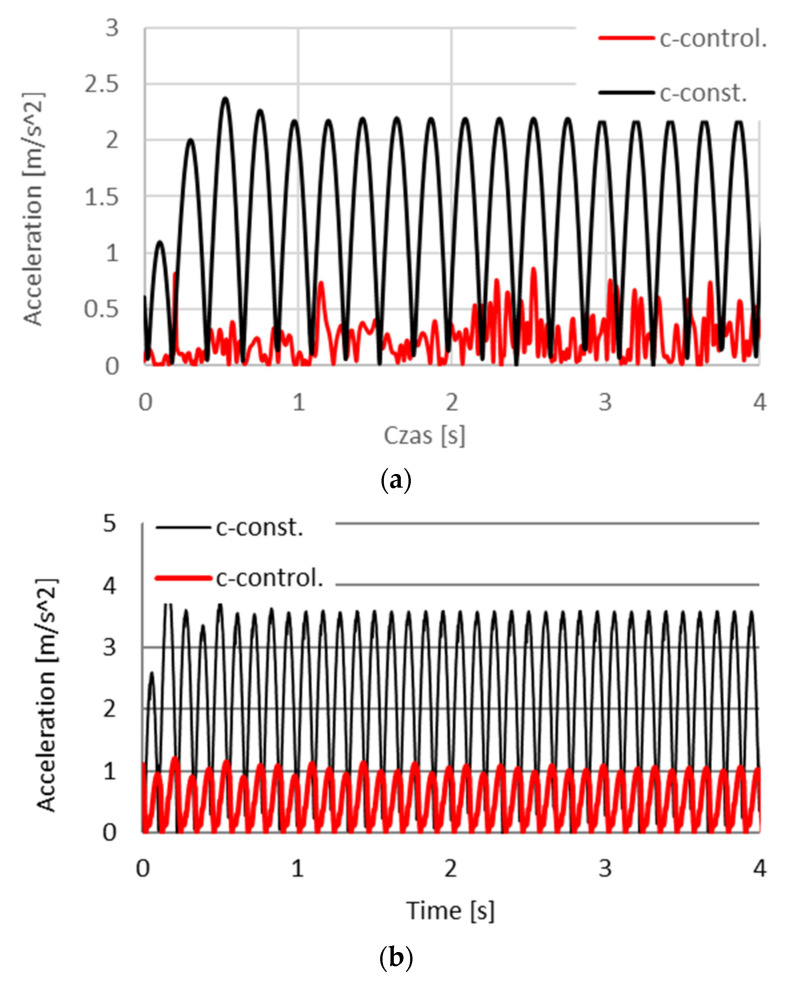
Changes of the module of accelerations based on numerical simulations of the model of the vehicle with the controlled PZ damper (c-control) and without control (c-const.), for frequency of excitation (**a**) 2.25 Hz and (**b**) 4.5 Hz.

A sample solution of the simulation of the module of accelerations in the function of time with a comfort criterion is shown in Figure 19a for excitation f = 2.25 Hz and in Figure 19b for excitation 4.5 Hz. One may observe in Figure 19a decrease of module of amplitudes of accelerations, with a constant damping coefficient from |a_max_| = 2.2 m/s^2^ to amplitude |a_max_| = 0.75 m/s^2^ in the system with controlled friction force T_i_ while in Figure 19b from |a_max_| = 3.6 m/s^2^ to amplitude |a_max_| = 1.05 m/s^2^ respectively. Evaluation of the influence of control of PZ dampers in the model of the vehicle performed, considering the comfort criterion, was conducted based on the norm ISO-2631, where three parameters were evaluated: comfort, inconvenience and harmfulness. From the results of studies one may conclude that the system with controlled friction force operates with lower amplitudes of accelerations. This is visible with harmonic excitations shown in Figure 19. Exposition time in comfort conditions, for velocity V_vehicle_ = 90 km/h was lengthened from 4 min to 9 h for simulation in Figure 19a and from 0 min to 9 h in Figure 19b. In the norm serving to evaluate vibrations, the value of amplitude as well frequency of the vibrations are considered. Vibrations in the range of vibrations harmful to a human (4–8 Hz) influences to the greatest extent the elongation of time of exposition.

## 6. Summary

The work presents the process of developing a controlled damper of vibrations, where to control forces a PZ valve with a piezoelectric element is used. Studies of properties of the controlled damper were conducted, where the voltage of the power supply to the piezoelectric stack was changed. The results of studies indicated that together with the voltage increase, the damping force increases.

In the prototype solution of the damper it is possible to change the range of the initial size of the gap, which has an influence on the initial value of the damping force. It also enables the change of the range of damping forces, which in turn enables the range of damping forces to be adjusted to a mechanical system.

Within the scope of the paper, the results of experimental investigations of the properties of the PZ damper in terms of the influence of temperature change were presented. Due to the fact that the PZ damper was filled with hydraulic oil, the temperature increase influenced the decrease in the value of damping forces.

The conducted experimental investigation serves to establish a mathematical model of the PZ damper as well as to estimate model parameters. Based on that, the results of simulation of the controlled damper were achieved and compared with experimental data. A high convergence of results of numerical studies and experimental studies was obtained. As a result, a method of identification parameters with criterion of evaluation of the obtained parameters of the model was proposed.

The developed model of the damper was used for further numerical investigations of the model of a vehicle equipped with controlled PZ dampers. The numerical simulation was conducted based on the developed model of a vehicle with the assumption of a comfort criterion. Based on the assumed criterion an effective algorithm of selection of values of damping forces was established. Analysis of results of studies indicates a definite elongation of exposition time in comfort conditions based on the norm ISO-2631. It was also possible to use the control algorithm with the criterion of wheel dynamic load change, where changes of dynamic load of a vehicle’s wheels were minimized.

The developed and presented methodology of studies may be applied in further studies related to improving transition properties of a vehicle using next generation dampers with a PZ valve. The developed original solution of a damper with a controlled PZ valve based on a piezoelectric stack may be a competitive solution for currently widespread dampers with a magneto-rheological fluid in terms of selected properties such as frictional wear, fluid segmentation or response time. PZ dampers have advantages such as short response time and the fact that they are based on the use of standard oil but they also have disadvantages such as the cost of piezoelectric materials, the characteristics of the piezoelectric element (force vs. stroke) and supply voltage different to that used, for example, in automotives. In addition, fast changes of regulation of the PZ damper require the use of appropriately fast power supplies of not so small power.

**Figure 20:**
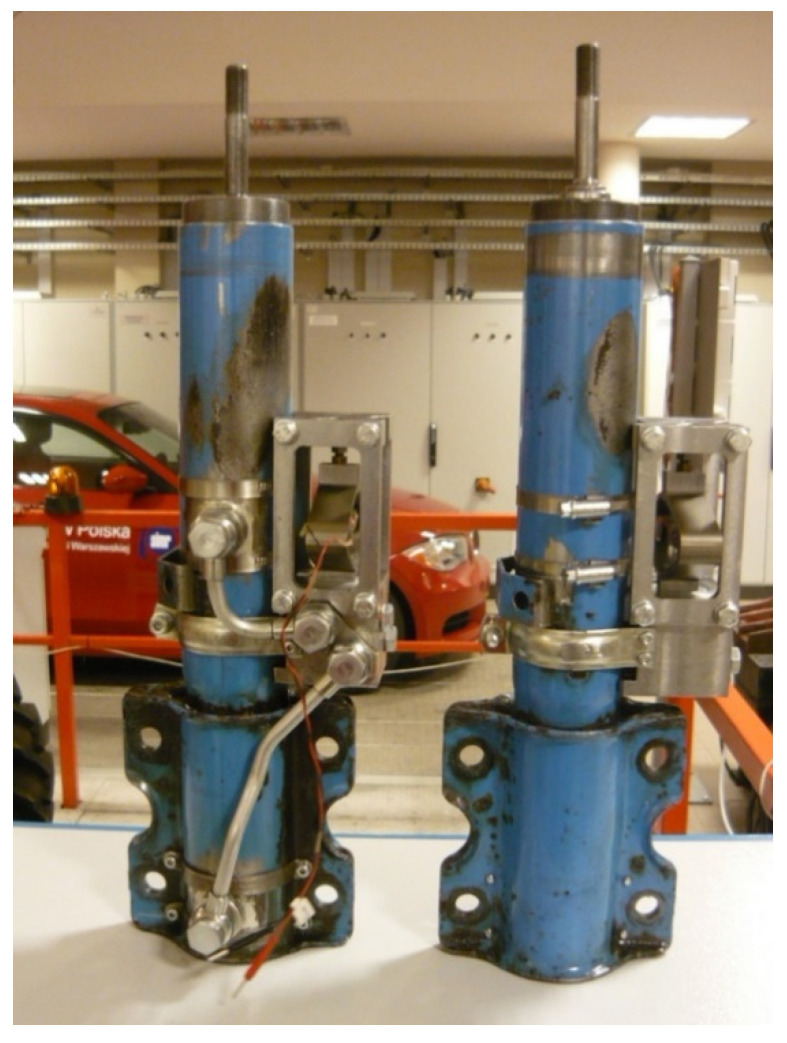
PZ damper—Ford Transit.

The original construction of the PZ damper and the results obtained serve to commence further research. A program of research is planned, related to developing a family of PZ dampers, which may be applied in different mechanical systems and aim to reduce vibrations of building structures. An experimental solution of a different type of the PZ damper, for a commercial vehicle, is shown in Figure 20.

It is also planned to verify the accuracy of operation of the assumed control algorithms in real conditions. Then, the developed construction solution and control algorithms may be used to conduct studies in the scope of limiting vibrations in other applications.

The results obtained may also serve to further develop the established system e.g., by using signals from other systems of a vehicle: steering, braking or drive system. For example, based on signals from steering, one may predict and change damping forces in the suspension of a vehicle. Such applications of controlled dampers will increase the safety level by limiting dynamic loads while driving on arches of a road as well as while accelerating and braking.

## Figures and Tables

**Table 1:** Values of the identified parameters in the model of the damper with the minimal gap.

Voltage [V]	T0 [N]	C [Ns/m]	k [N/m]
0	820	0.92 × 10^4^	5.81 × 10^5^
100	1985	1.64 × 10^4^	7.78 × 10^5^
150	3410	1.37 × 10^4^	11.10 × 10^5^

**Table 2:** Values of the identified parameters of the model of the damper with minimal gap (temperature 22 °C).

Voltage [V]	T0 [N]	C [Ns/m]	k [N/m]
0	301	2.72 × 10^3^	1.99 × 10^5^
50	356	3.30 × 10^3^	3.58 × 10^5^
100	796	3.04 × 10^3^	3.75 × 10^5^
150	1325	2.56 × 10^3^	4.23 × 10^5^

**Table 3:** Values of identified parameters in the model of the damper with minimal gap (temperature 43 °C).

Voltage [V]	T0 [N]	C [Ns/m]	k [N/m]
0	169	3.30 × 10^3^	2.14 × 10^5^
50	190.6	2.59 × 10^3^	2.61 × 10^5^
100	1363	2.66 × 10^3^	3.46 × 10^5^
150	1078	3.38 × 10^3^	3.85 × 10^5^

## Data Availability

The data presented in this study are available on request from the corresponding author.
